# Synergistic stabilization of a menthol Pickering emulsion by zein nanoparticles and starch nanocrystals: Preparation, structural characterization, and functional properties

**DOI:** 10.1371/journal.pone.0303964

**Published:** 2024-06-06

**Authors:** Minghe Yang, Shujin Cheng, Lei LÜ, Zhonghui Han, Jinxing He

**Affiliations:** 1 School of Food Science and Engineering, Qilu University of Technology (Shandong Academy of Sciences), Jinan, China; 2 School of Bioengineering, Qilu University of Technology (Shandong Academy of Sciences), Jinan, China; 3 General Tobacco Group Co., Ltd, Jinan, China; University of Education, Lahore, PAKISTAN

## Abstract

A Pickering emulsion was synergistically stabilised with zein nanoparticles (ZNPs) and starch nanocrystals (SNCs) to prepare it for menthol loading. After response surface optimisation of the emulsion preparation conditions, a Pickering emulsion prepared with a ZNPs:SNCs ratio of 1:1, a particle concentration of 2 wt% and a water:oil ratio of 1:1 provided the highest menthol encapsulation rate of the emulsions tested (83%) with good storage stability within 30 days. We examined the bilayer interface structure of the emulsion by optical microscopy, scanning electron microscopy, and confocal laser scanning microscopy. The results of simulated digestion experiments showed that the release rate of free fatty acid was 75.06 ± 1.23%, which ensured bioavailability. At the same time, the emulsions facilitated the slow release of menthol. Bacteriostatic studies revealed that the Pickering emulsion had a protective effect on menthol, with the most significant inhibitory effects on *Escherichia coli* and *Staphylococcus aureus* under the same conditions. Overall, this study proposes a novel approach for the application and development of l-menthol by combining it with Pickering emulsion.

## 1. Introduction

l-Menthol (C_10_H_20_O; (1R,2S,5R)-2-isopropyl-5-methylcyclohexanol) is a naturally occurring, chiral, volatile, cyclic terpene alcohol. It is extracted primarily from the peppermint plant (*Mentha* × *piperita*; family Lamiaceae), and has a characteristic minty odor [1]. Most of the current studies on emulsion systems are based on peppermint essential oils. For example, Mohammadifar et al. [2] studied the effects on osteoarthritis of nanoemulsions loaded with peppermint and rosemary essential oils. Zhu et al. [3] successfully prepared peppermint oil nanoemulsions using soy protein isolate and phosphatidylcholine as emulsifiers. However, peppermint essential oil has many components and is commercially available in different formulations. Menthol, the main constituent of peppermint essential oil, has a wider range of applications than peppermint essential oil. However, the use of menthol is limited owing to its high volatility, instability at high temperatures, and short shelf life [4], and its encapsulation in emulsion systems has not been extensively investigated. Emulsion systems can expand the range of applications for menthol, reduce its loss during application, and maximise its use.

The reduction in the quantity of active substances caused by the increase in biological activity and the delivery of active agents to specific drugs, so different emulsion formulations are often used to load different active ingredients [5]. Microemulsion systems are optically isotropic, transparent, and thermodynamically stable homogeneous dispersions of two immiscible fluids, usually oil and water [6], that can be used in areas such as medicine and pesticides [7]. For example, Nazar et al. [8] prepared a novel oil-in-water (o/w) microemulsion comprising castor oil, Tween 80, ethanol, and a phosphate buffer for the enhanced loading of the anti-inflammatory drug piroxicam. Salim et al. [9] developed a new o/w microemulsion (μE)-based soft template to prepare nanoparticles of the poorly water-soluble drug irbesartan. The microemulsion improved the dissolution rate of the drug. Nazar et al. [10] prepared a novel aqueous oil-biocompatible μE formulation comprising clove oil, Tween 20, 2-propanol, and water to encapsulate the antibiotic levofloxacin. Unlike microemulsions, Pickering emulsions are stabilized with food-grade colloidal particles, and are therefore often used to encapsulate nutrients.

Zein nanoparticles (ZNPs) are prepared from zein by antisolvent precipitation, and retain zein’s oleophobic and hydrophobic properties. They are also commonly used as stabilizers for Pickering emulsions [11]. However, Pickering emulsions prepared from ZNPs are only stable for approximately 2 weeks under alkaline conditions. To produce stable emulsions, it is necessary to decrease the hydrophobicity of the zein particles by incorporating hydrophilic components [12]. The crystalline region of starch that remains after the removal of the amorphous region comprises starch nanocrystals (SNCs). The surfaces of SNCs are rich in hydroxyl groups. The particles are highly hydrophilic and aggregate when dispersed in water because they form hydrogen bonds. Therefore, they are often used to make Pickering emulsions [13]. Importantly, SNCs can be prepared from natural starches from a variety of sources by mild acid hydrolysis [14]. There have been numerous studies of SNCs from various sources. For example, Azfaralariff et al. [15] found that *Metroxylon sagu* starch has high potential as a new source of SNCs, and Pickering emulsions prepared from sago SNCs are highly stable.

In the present paper, we report a novel way of ensuring the protection and slow-release of l-menthol from an emulsion by embedding the l-menthol in a Pickering emulsion combined with l-menthol. Our aim was to mitigate the shortcomings of l-menthol, which is unstable, thereby expanding its scope of application. Moreover, the emulsion ameliorates the irritant effect of l-menthol, allowing its useful antioxidant and antimicrobial properties to take effect. This will extend its usefulness to the food industry.

## 2. Materials and methods

### 2.1. Materials

The l-menthol (purity ≥ 99%) was obtained from Beijing Coolaber Technology Co., LTD. Waxy maize starch (food grade) was obtained from Tianjin Wuxi Tianyu Biotechnology Co. The maize alcohol soluble protein (food grade) was from Sigma-Aldrich. The soya bean oil (reagent grade) was from Shanghai Macklin Co. The malondialdehyde (MDA) content assay kit was from Beijing Solarbio Science & Technology Co., Ltd. Simulated gastric fluid, simulated salivary fluid, and simulated intestinal fluid were purchased from Beijing Coolaber Technology Co., LTD. Oxalate, hydrochloric acid, caustic soda, sodium chloride, and anhydrous ethanol were obtained from Sinopharm Chemical Reagent Co., Ltd (Shanghai, China). All chemicals and drugs were of analytical grade.

### 2.2. Methods

#### 2.2.1 Preparation of ZNPs

We dissolved 1 g of zein powder in 30 mL of aqueous ethanol (80 vol%) and stirred the solution for 30 min. Next, we placed 70 mL of water in a three-necked flask, and slowly added the zein solution while stirring mechanically at 500 rpm for 2 h. After mixing, we removed the ethanol by rotary evaporation at 40°C to obtain a dispersion of the ZNPs. Subsequently, we obtained a ZNP powder by lyophilization.

### 2.3 Preparation of the SNCs

We dissolved 50 g of waxy maize starch in 500 mL of H_2_SO_4_ (3.16 M) and mechanically stirred the solution at 200 rpm in a water bath at 40°C for 7 days. Next, we centrifuged the mixture at 8000 *g* for 10 min to remove the supernatant, and washed the bottom sediment with deionized water. The steps described above were repeated until the pH of the supernatant was close to neutral. An SNC powder was obtained by lyophilization.

### 2.4 Preparation of the Pickering emulsion

The pH values of the SNC and ZNP dispersions were adjusted to 4 with 1 M HCl or NaOH. We then dissolved 40 mg/ml l-menthol in soya bean oil. Next, we mixed particle dispersions of 2 wt% SNCs and 2 wt% ZNPs in a 1:1 ratio with the soya bean oil, and homogenized the mixture for 2 min at 17,000 rpm to obtain the final o/w Pickering emulsion.

### 2.5 Response surface experiment design

Based on a one-factor experiment, Response Surface Optimisation was chosen to create, refine and optimise a problem where the output factor (composite score) was influenced by several input factors (pH, particle concentration, particle-liquid ratio) [16]. We designed a three-factor, three-level experiment using pH (X_1_), particle liquid ratio (X_2_), and particle concentration (X_3_) as the corresponding factors, and the combined scores of emulsification value (Y_1_), potential (Y_2_), and embedding rate (Y_3_) as response values, as shown in Table 1.

**Table 1:** Experimental table of three factors and three levels.

Level	pH	Proportion of particle liquid	Particle concentration
-1	3	1:2	1wt%
0	4	1:1	2wt%
1	5	2:1	3wt%

The weight of emulsification value (Y_1_), potential (Y_2_), and embedding rate (Y_3_) were calculated by principal component analysis, and the membership degree of 3 indices was calculated using the membership degree calculation formula to obtain the composite score of the emulsion. Based on the single-factor experiments, 17 groups of response surface tests were designed using Design Expert 8.0.6.1 software. The test design and results are shown in Table 2. The membership degree and composite score were calculated according to the S1 Text.

**Table 2:** Design of experiment matrix for pH, proportion of particle liquid and particle concentration.

Run	pH	Proportion of particle liquid	Particle Concentration	Composite Score
1	5	0	3	0.449
2	5	1	2	0.213
3	4	0	2	0.754
4	3	0	3	0.681
5	4	0	2	0.674
6	4	-1	3	0.499
7	3	-1	2	0.454
8	4	1	3	0.343
9	4	-1	1	0.347
10	4	0	2	0.758
11	5	0	1	0.350
12	4	0	2	0.684
13	5	-1	2	0.313
14	4	0	2	0.655
15	3	1	2	0.503
16	4	1	1	0.273
17	3	0	1	0.436

#### 2.5.1 Gas chromatography conditions

We determined the l-menthol content by gas chromatography using the People’s Republic of China Tobacco Industry Standard. The gas chromatography parameters were as follows: the capillary column dimensions were 30 m × 0.25 mm × 0.25 μm; the stationary phase comprised crosslinked polyethylene glycol; the inlet temperature was 250°C; the starting temperature was 160°C for 2 min; the programmed temperature rose from 160 to 200°C at a rate of 5°C /min; the detector temperature was 250°C; the carrier gas comprised a constant current of highly pure nitrogen or helium (1.2 mL/min); the auxiliary gas comprised air (400 mL/min), high-purity nitrogen (40 mL/min), high-purity nitrogen or helium (25 mL/min); the split ratio was 50:1; and the sample size was 1.0 μL. Using the conditions described above, the total analysis time was 10 min.

#### 2.5.2 Production of standard curves

We weighed approximately 0.5 g of l-menthol to the nearest 0.0001 g, dissolved it in an extractant comprising anhydrous ethanol solution containing n-propyl benzoate with a concentration of 0.2–0.3 mg/mL, transferred it to a 100 mL volumetric flask, and adjusted the volume to prepare a standard reserve solution. We then diluted the standard reserve solution with extractant to produce a solution with a concentration of 0.05–0.5 mg/mL. The L-Menthol embedding rate of the Pickering emulsion was calculated according to the S2 Text.

The curve of the working standard solution was determined, the l-menthol and internal standard ratio in each solution was calculated, and the linear regression equation describing the relationship between the concentration of l-menthol and the peak area ratio was obtained. Next, 2 mL of the emulsion was added to 5 mL of anhydrous ethanol. The content of l-menthol was determined after 40-fold dilution of the supernatant.

### 2.6 Characterization of the Pickering emulsion

#### 2.6.1 Particle size and zeta (ζ)-potential of the emulsion

The particle size distribution and ζ-potential of the Pickering emulsion samples were measured using a Malvern particle size analyzer. Specifically, each emulsion sample was diluted 150-fold before measurement. At 25°C, the refractive index and absorption index of soybean oil are 1.45 and 0.001, respectively, and the refractive index and absorption index of water are 1.33 and 0, respectively [17]. Each sample was analyzed more than three times, and the results were the averages of three or more measurements.

#### 2.6.2 Microstructure of the Pickering emulsion

We removed 100 μL of the emulsion and placed it in a 1.5 mL centrifuge tube, added 1 mL of deionized water to dilute it, removed 100 μL of the resulting liquid, dropped it onto a microscope slide, covered it, and obtained microscopic photographs of the emulsion using a microscope.

A 0.1 wt% fluorescent whitening agent was added to the SNC dispersion, and a dyed dispersion was obtained after magnetic stirring for 12 h in the dark. The emulsion samples were then prepared according to the method described in Section 2.4 and 40 μL of Nile Blue A solution was added to each (0.1 wt%). Isopropyl alcohol solvent and 1 mL of each emulsion were mixed. The appropriate emulsion sample was then evenly applied to a 35 mm confocal laser petri dish, which was placed on the stage of a confocal laser scanning microscope. The excitation wavelengths were 633 and 405 nm, the scanning density was 1024 × 1024. The fluorescence images of the emulsion were collected.

The Pickering emulsion was prepared using 50% n-hexane instead of the oil phase according to the method described in Section 2.4. The freeze-dried powder of the emulsion was smeared on the sample table, and gold sputtering was carried out using an ion-sputtering apparatus with the current set to 20 mA and the time set to 2 min. The surface of the starch particles was examined using a scanning electron microscope at 10 kV.

#### 2.6.3 Rheological measurements

The rheological properties of the Pickering emulsion at room temperature were determined using an Anton Paar rheometer (Anton Paar, Australia) [18]. A PP50 parallel flat probe with a radius of 25 mm was used in all tests, and the gap was set to 1 mm. The linear viscoelastic range of the Pickering emulsion samples was determined by strain scanning experiments at 1 Hz and a strain range of 0.01%–100%. Frequency scanning experiments (0.1–100 Hz) were carried out in the linear viscoelastic range, and the energy storage modulus (G′) and loss modulus (G″) of each emulsion sample were determined. The apparent viscosity of each emulsion was measured at shear rates of 0.1–100 s^-1^).

The temperature tests were carried out using a PP50 parallel plate probe with a radius of 25 mm and a gap of 1 mm, and the temperature rise and fall rates were 5°C/min.

#### 2.6.4 Determination of the antioxidant capacity of the emulsion

The antioxidant capacity of the emulsion was evaluated by determining the content of MDA, and the content of the secondary oxide of MDA was determined by the thiobarbituric acid method [19]. Absorbance values were measured and calculated at 600, 532, and 450 nm according to the instructions provided with the MDA kit.

#### 2.6.5 *In vitro* digestive simulation

According to previous reports [20], we mixed 5.0 mL of each emulsion, 3.5 mL of simulated salivary fluid, 0.5 mL of α-amylase solution (1500 U/mL), and 1 mL of ultrapure water. The pH of each mixture was then rapidly adjusted to 7.0, and the mixture was incubated in a shaker at a constant speed of 100 rpm for 5 min at 37°C to simulate the environmental conditions associated with chewing food in the mouth and to initiate digestion.

Next, we mixed 10.0 mL of each orally digested sample, 7.5 mL of simulated gastric fluid, 1.6 mL of porcine pepsin (25,000 U/mL; mucin concentration: 1.5 mg/mL), 0.2 mL of HCl (1.0 M), and 0.7 μL of ultrapure water. The pH of each mixture was then rapidly adjusted to 2.0, and the digestion process was carried out for 2 h in a shaker at 37°C while stirring at a constant speed of 100 rpm to simulate the gastric environment.

Next, we mixed 20.0 mL of each gastrically digested sample, 11.0 mL of simulated intestinal fluid, 5.0 mL of trypsin solution (200 U/mL), 2.5 mL of bile salts (0.684 mg/mL), 40 μL of calcium chloride solution (0.3 M), and 1.31 mL of ultrapure water. The pH of the mixture was then rapidly adjusted to 7.0, and the digestion process was carried out in a shaker at a constant speed while stirring at 100 rpm for 2 h at 37°C to simulate the environment of the small intestine. The pH of the digestion solution was maintained at 7.0 by the constant addition of 0.1 M sodium hydroxide during this period, and the release of fatty acids from the various emulsion samples was evaluated based on the consumption of sodium hydroxide. The FFA release rate was calculated according to S3 Text.

#### 2.6.6 Bacteriostatic testing of the Pickering emulsion

Beef paste–peptone liquid medium was inoculated with drug-resistant *E*. *coli* and *S*. *aureus*, and the samples were incubated in shaking flasks. The samples were incubated at 37°C overnight while shaking at 200 rpm. The next day, each bacterial suspension was re-inoculated into a new beef paste–peptone liquid medium at a ratio of 1:40, and the mixture was incubated for approximately 3 h in accordance with the culture conditions described above, during which time the bacteria were in the logarithmic phase [21].

We inoculated the logarithmic phase bacterial suspension into a new beef paste–peptone liquid medium at a ratio of 1:40, placed it on a shaker, and incubated it for 12 h at 37°C while shaking at 200 rpm. An l-menthol Pickering solution and an l-menthol solution of the same concentration were used as the drug group, a Pickering emulsion without embedded l-menthol was used as the control group, and a bacterial suspension without any drug was used as the blank group. Bacterial suspensions from each blank, control, and drug group were gradient diluted and 150 μL of each was applied to beef paste–peptone solid medium. After 12 h of incubation in the incubator, the number of bacterial colonies on the solid plate from each group was counted (colony-forming unit (CFU) method). The experiment was repeated three times to obtain the average value [22].

### 2.7 Statistical analysis

The results are presented as the mean and standard deviation based on the three duplicate samples. Statistical analysis was performed using one-way analysis of variance (ANOVA) in SPSS (version 22; IBM Corp., New York, USA) with a significance level of P < 0.05. Graphs were created using the Origin Pro 8.5 program (Origin Lab, Massachusetts, USA). The response surface tests were analyzed by linear regression and ANOVA (P < 0.05) using Design Expert (Version 8.0.6.1).

## 3. Results and discussion

### 3.1 Determination of the embedding rate

**Fig 1:**
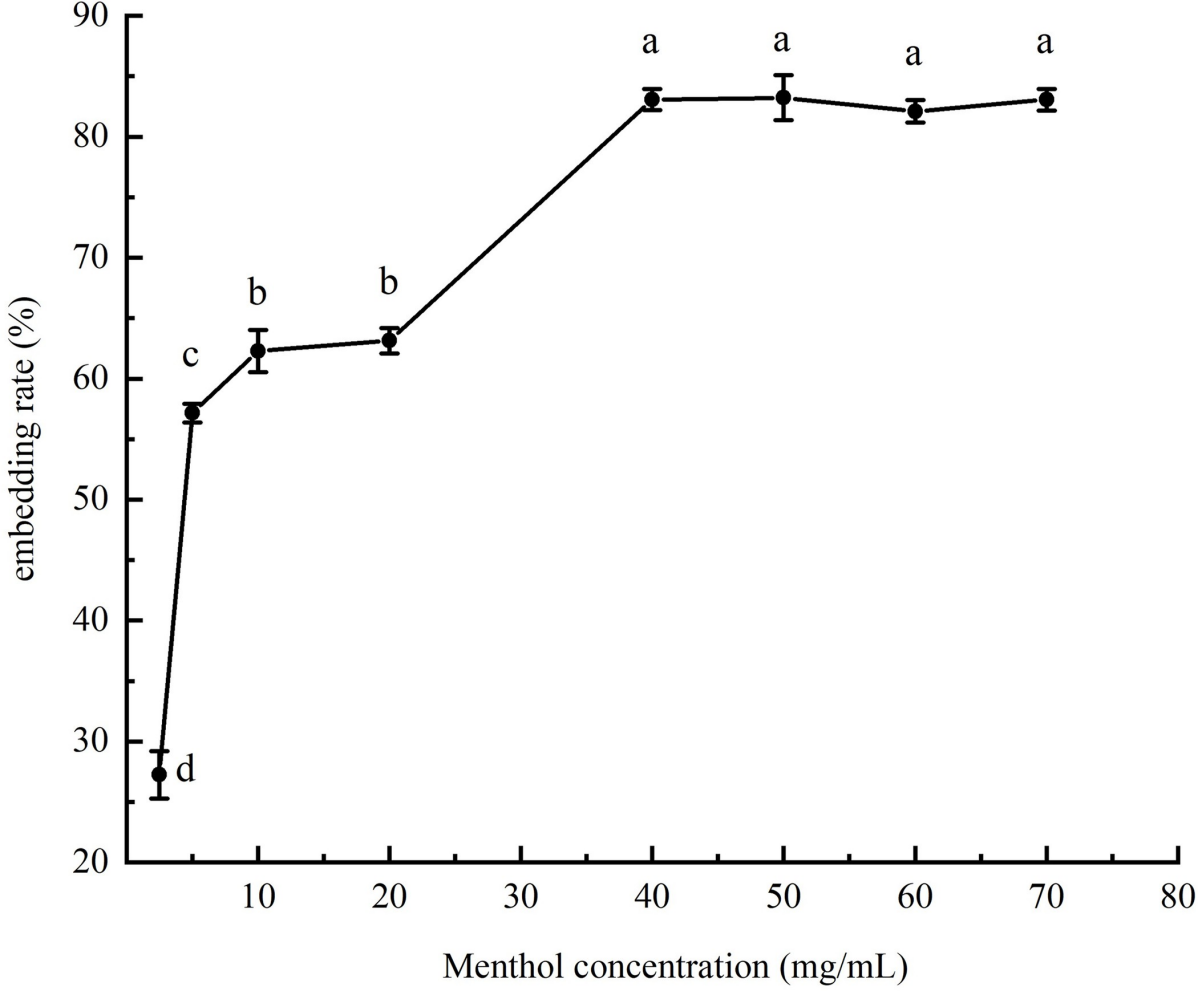
Relationship between menthol concentration and embedding rate, different letters (a-e) indicate significant differences (P < 0.05).

**Fig 2:**
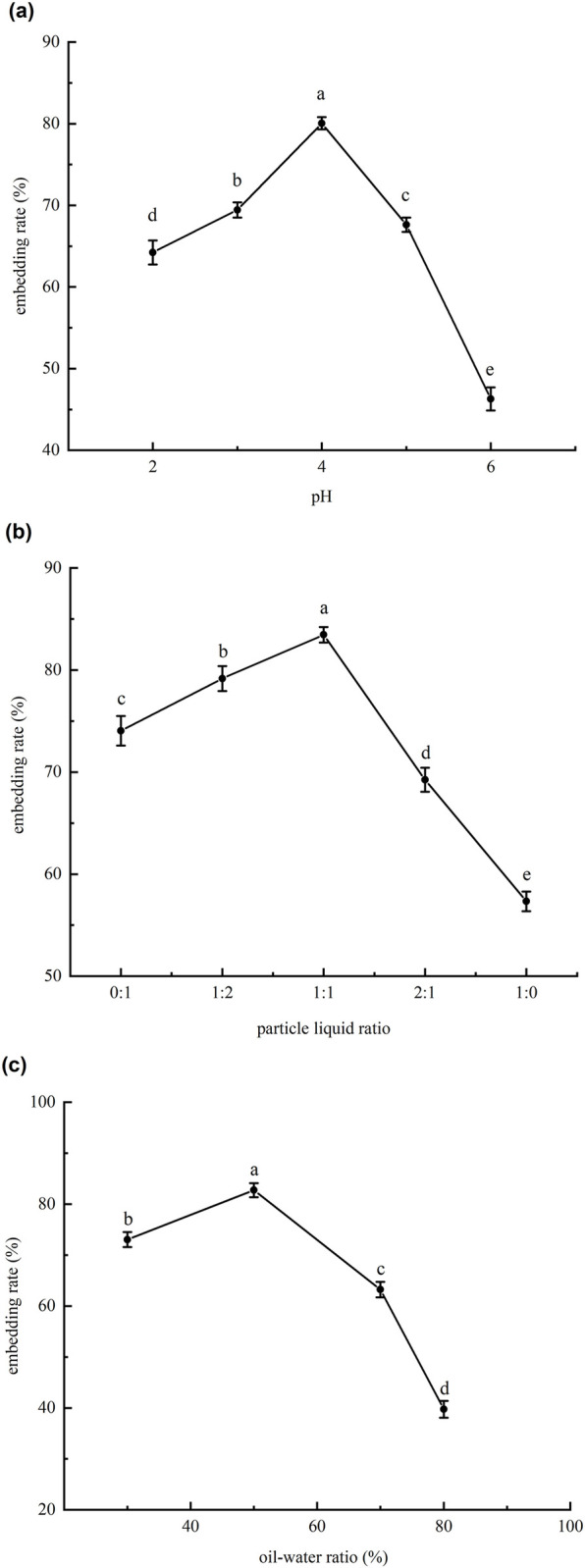
(a) (b) (c) were graphs of pH, particle liquid ratio and oil/water ratio (%) versus embedding rate, different letters (a-d) indicate significant differences (P < 0.05) among the different groups and the error bars represent the SD.

The standard gas chromatography curve of l-menthol in emulsion was described by y = 3.03377x + 0.02222. The correlation coefficient (R^2^) for the l-menthol standard curve was 0.99984, indicating a good fit. The amount of l-menthol added was determined by eight single factorial experiments with different l-menthol concentrations and the results of the embedding rate are shown in Fig 1 among the different groups and the error bars represent the SD). When the l-menthol concentration was increased to 40 mg/mL, the encapsulation rate was 83% and there was no significant increase in the encapsulation rate of the Pickering emulsion as the l-menthol concentration was further increased. This implies that the addition of l-menthol at that point was excessive and that the soybean oil was saturated with l-menthol. Therefore, 40 mg/mL was selected as the optimum concentration of l-menthol in the experiment. The relationship between the embedding rate and each factor is shown in Fig 2(A)–2(C). An appropriate level was selected for the response surface experiment based on the Box–Behnken combination design.

### 3.2 Particle size and ζ-potential

The particle size and ζ-potential of a Pickering emulsion are essential indices that are measured to assess the stability of an emulsion [23, 24]. The stability of a Pickering emulsion increases as the particle size decreases [25]. A higher ζ-potential indicates greater stability without any aggregation [26]. Furthermore, a negative ζ-potential indicates high colloidal stability, and the absolute values of the potentials of the stable emulsions were all ≥ 30 mV owing to electrostatic repulsion between the nanoparticles [27, 28]. The potential and particle size of the blank Pickering emulsion and the menthol Pickering emulsion are given in Table 3. The droplet size of the Pickering emulsion decreased with the addition of l-menthol because the hydrogen bonding force between the solid particles was reduced, preventing aggregation and crystallization on the surface of the emulsion droplet. The absolute potential values of the emulsions were all approximately 30 mV, which suggests that the emulsions had excellent storage stability.

**Table 3:** Particle size and potential of emulsion of the blank and the menthol Pickering emulsions.

Sample	Particle size (μm)	Zeta potential (mV)
Blank Pickering Emulsion	32.47±2.76	-37.27±2.19
Menthol Pickering Emulsion	33.09±4.51	-35.34±1.86

### 3.3 Analysis of response surface experimental data

The regression model of the response surface is presented in Table 4, with P < 0.01, indicating that the model achieved a highly significant level. The probability of the F value of this intensity being influenced by random interference was only 0.02%. The misfit term (P = 0.4488) was greater than 0.05, indicating that it was not significant. The coefficient of determination of the regression model, R^2^ = 0.9668, indicated that the experimental data fitted the regression equation well and the experimental error was small. Therefore, the regression model was considered reliable and could be used to analyze and predict the preparation of the menthol Pickering emulsion. A 95% confidence interval was chosen to assess the significance of the model. All models were determined on the basis of the highest level of additional variables required and no jaggies were present in the RSM program [16]. ANOVA indicated that all factors in the secondary term had highly significant effects on the composite score of the emulsion index (P < 0.01), and the order of factors influencing the composite score in the primary term was X_3_ (particle concentration) > X_1_ (pH) > X_2_ (particle liquid ratio).

**Table 4:** ANOVA by RSM for the pH, particle liquid ratio, and particle concentrations and rut depth for the menthol Pickering emulsion.

Source	Sum of squares	df	Mean square	F-Value	P-value	Observation
Model	0.48	9	0.054	22.66	0.0001	significant
X_1_-pH	0.070	1	0.070	29.62	0.0010	
X_2_-Proportion of particle liquid	9.870E-003	1	9.870E-003	4.17	0.0805	
X_3_-particle concentration	0.040	1	0.040	16.91	0.0045	
X_1_X_2_	5.329E-003	1	5.550E-003	2.34	0.1696	
X_1_X_3_	5.329E-003	1	5.329E-003	2.25	0.1772	
X_2_X_3_	1.681E-003	1	1.681E-003	0.71	0.4273	
X_1_^2^	0.051	1	0.051	21.66	0.0023	
X_2_^2^	0.21	1	0.210	89.13	<0.0001	
X_3_^2^	0.056	1	0.056	23.77	0.0018	
Residual	0.017	7	2.368E-003			
Lack of Fit	7.462E-003	3	2.487E-003	1.09	0.4488	Not significant
Pure Error	9.112E-003	4	2.278E-003			
Cor Total	0.50	16				
			R^2^ = 0.9668	R^2^adj = 0.9242		

df: degree of freedom, F-values: Fisher-statistical test values, p-values: probability values, LoF: lack of fit.

The RSM ANOVA model is presented in Table 5. The “Pred R-Squared” value was 0.7325, which was essentially consistent with an “Adj R-Squared” value of 0.9242, i.e., the difference was less than 0.2, indicating good predictability. The R^2^ value was high and almost constant, indicating that the quadratic model fitted the actual data well. In addition, the standard deviation of the model was significantly smaller than the obtained mean, suggesting the suitability of ANOVA. The smaller the standard deviation of the mean of the resulting model, the smaller was its variance with the test data, implying that less uncertainty was introduced into the model by the experimental results [16]. In light of the above analysis, it can be concluded that the generated model is suitable for modelling, optimizing, and predicting the manufacture of a menthol Pickering emulsion.

**Table 5:** Regression analysis values for the responses.

Statistical analysis	Composite Score	Comment
Standard deviation	0.049	The models are significant fornavigation around the designspace
Mean correlation	0.49	
coefficient	0.9668	
Adj.R^2^	0.9242	
Pred.R^2^	0.7325	
Adequate precision	13.404	
Coefficient of variance	9.86	

**Fig 3:**
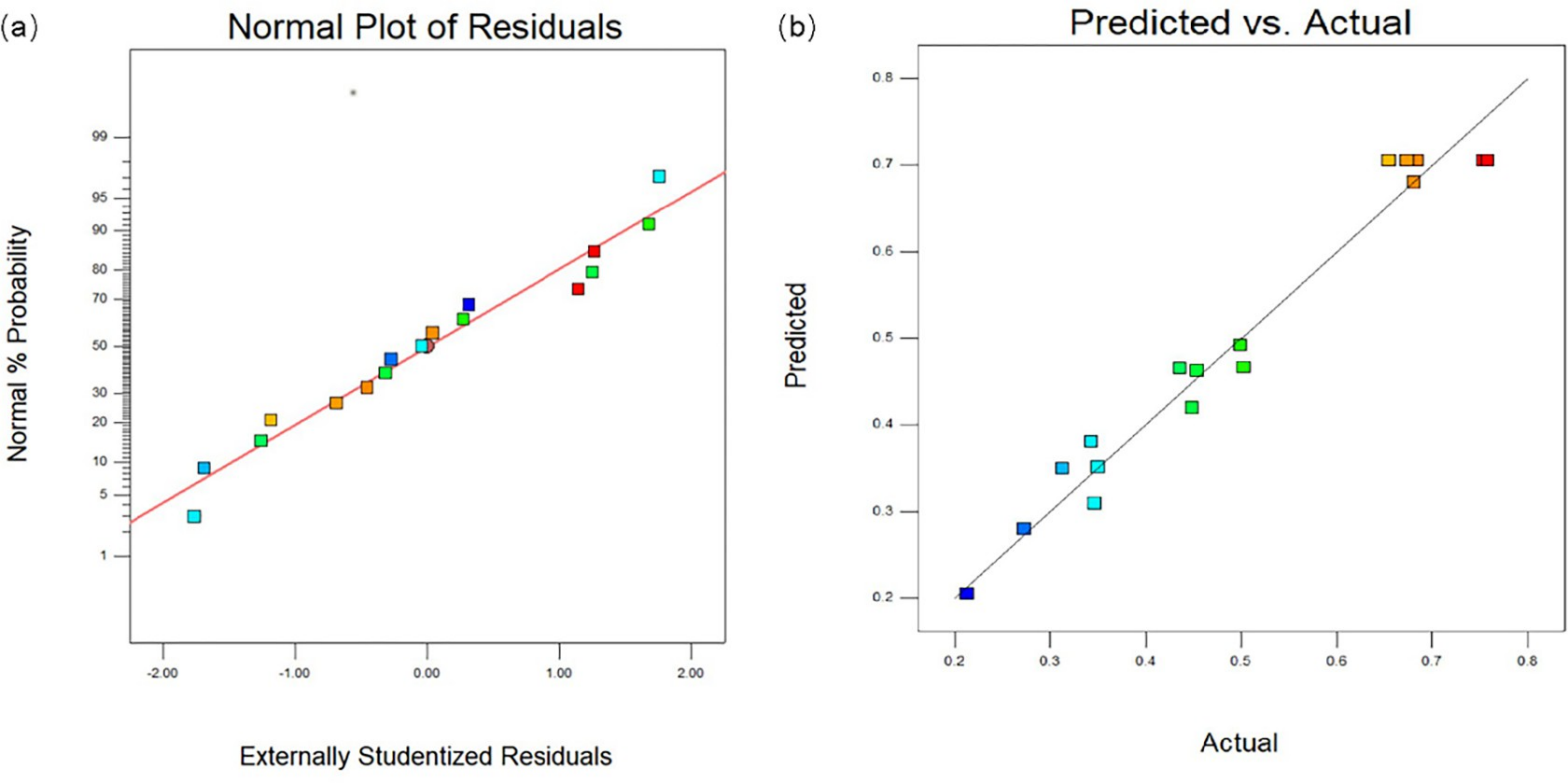
(a) were normal plot of residuals, (b) were predicred vs. actual.

A normal plot of residuals of the composite score is presented in Fig 3(A). The figure indicates a close fit between the residual handicap and a straight line. This observation verifies the validity of the regression model because the data fall within the range of equal lines. The predicted and actual laboratory values used to evaluate the fit and accuracy of the model are shown in Fig 3(B). The symmetrical distribution of the data points around the 45° line indicated a close fit between the model and the experimental data.

**Fig 4:**
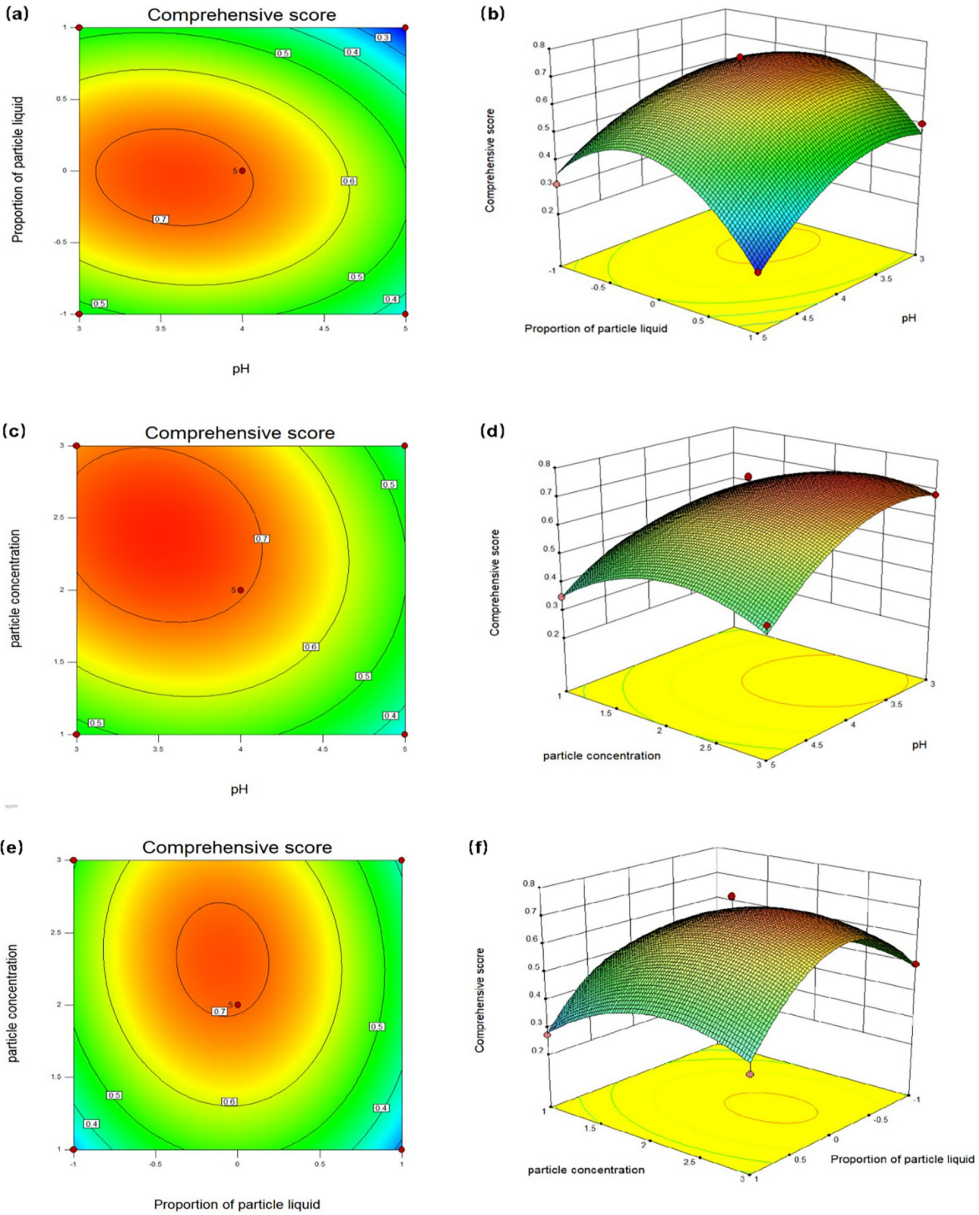
2-D and 3-D RSM surface showing the stimulatory influence of pH, Particle liquid ratio and Particle concentration on Composite score, (a) and (b) were the stimulatory influence of pH and Particle concentration on Composite score; (c) and (d) were the stimulatory influence of pH and Particle liquid ratio on Composite score; (e) and (f) were the stimulatory influence of Particle liquid ratio and Particle concentration on Composite score.

Graphs and contour plots are displayed in Fig 4(A) and 4(B). They illustrate the influence of pH and the Proportion of particle liquid on composite scores. The surface plot revealed that as the “Proportion of particle liquid” was changed from -1 to 1, the composite score initially increased to a certain value and subsequently decreased. This was owing to the poor stability of Pickering emulsions prepared from either ZNCs or SNCs alone. Higher stability was achieved in Pickering emulsions that were synergistically stabilized by mixing the two in a certain ratio [29–31].

The plot and contour diagram of the composite scores are shown in Fig 4(C) and 4(D). They reveal the influence of pH and particle concentration. The contour diagram shows that as the particle concentration increased from 1 to 3 wt%, the contour became curved and the composite score decreased after reaching a certain value. This was because as the concentration of colloidal particles increased, more colloidal particles could be adsorbed at the oil–water interface, which reduced the droplet size of the prepared Pickering emulsion. Therefore, the physical barrier formed by these colloidal particles around a droplet stabilized the emulsion [32]. When the concentration of colloidal particles continued to increase, the excess particles could not be adsorbed at the interface but remained free in the continuous phase. The particles were able to form a three-dimensional network structure in the continuous phase [33]. Although the stability of the emulsion increased, the embedding rate of l-menthol was affected by the three-dimensional network structure and therefore decreased. X_2_ (proportion) and X_3_ (particle concentration) appear flat and oval in the contour plots of the composite score emulsion index in Fig 4(E) and 4(F), and the response surface figure is steep. This indicates significant X_2_–X_3_ interaction.

### 3.4 Storage stability

The newly prepared emulsion was sealed in a 15 mL sample bottle at 25°C, and the emulsification index of the emulsion was determined every 5 days. The storage stability of the Pickering emulsion at room temperature was determined by measuring the emulsification index (CI, %), which was calculated using the following equation:

$$CI=(\frac{v_{2}}{v_{1}})\times 100\%$$

where, v_2_ is the emulsion phase volume (mL) and v_1_ is the sample volume (mL).

**Fig 5:**
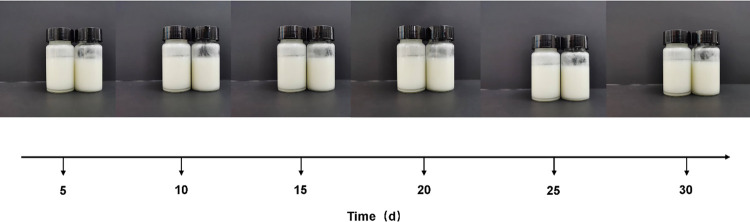
Changes in emulsification values of Pickering emulsions at different storage times, A was the blank emulsion, B was the menthol Pickering emulsion.

We sampled the blank and menthol Pickering emulsions every 5 days, and the results are shown in Fig 5. The figure reveals that the stability of the emulsion was minimally affected by the addition of l-menthol extract. Furthermore, the emulsification index of the emulsion remained at 100% after storage for 1 month. Therefore, the prepared Pickering emulsion was able to maintain its stability without phase separation over long periods at room temperature. The ternary system of O/W emulsions is very stable, Muhammad Faizan Nazar et al. [34] developed two microemulsion formulations for loading the antimycobacterial drug mirabilone (MBG) and the characterisation results showed that the emulsions were stable over a storage period of 7 months.

### 3.5 Microstructure of the emulsion

#### 3.5.1 Optical microscopy

**Fig 6:**
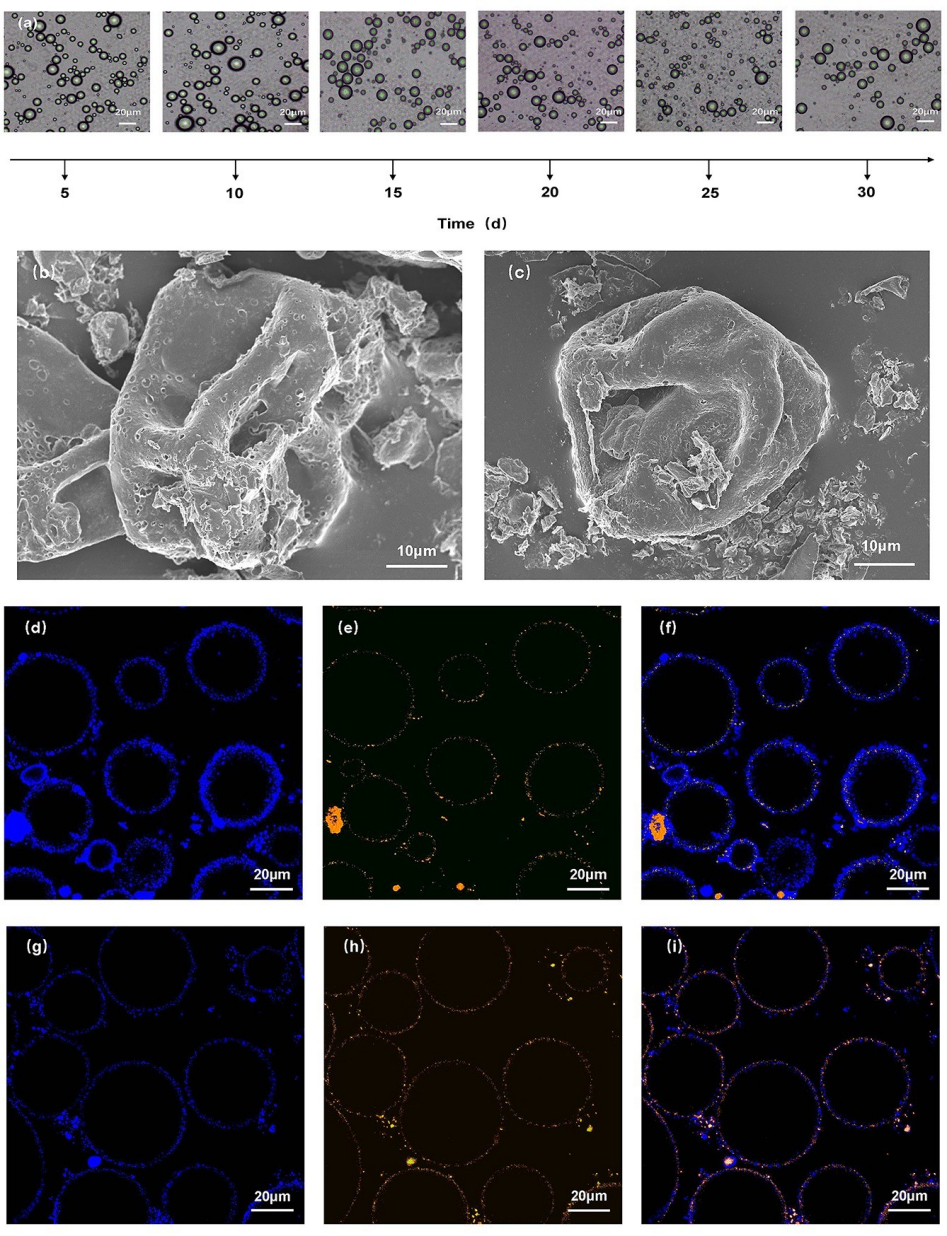
Optical microscopy, scanning electron microscopy and laser confocal microscopy diagrams of Pickering emulsion, (a) was the optical microscopy images of the menthol Pickering emulsion during storage at a scale of 20 μm,(b) and (c) were the SEM images of the menthol Pickering emulsion and the blank Pickering emulsion, and the magnification of (b) and (c) were 1000 times, (d) to (i) were the CLSM images of the menthol Pickering emulsion and the blank Pickering emulsion.

To verify the morphological structure of the oil droplets during storage, and to further demonstrate the stability of the emulsion, the menthol Pickering emulsion was selected during storage and examined under a light microscope. As shown in Fig 6(A), the oil droplets in the emulsion retained their shape after 1 month of storage, and the number of oil droplets under the eyepiece decreased by approximately 7%–8%, demonstrating the stability of the emulsion.

#### 3.5.2 Scanning electron microscopy

The liquid phase of the emulsion was removed by freeze-drying, and the shell structure formed by the solid particles was examined. The solid shell membrane structure is illustrated in Fig 6(B) and 6(C). This observation confirms the mechanism of stable particle stabilization in the emulsion.

The l-menthol crystals melted in the oil phase and were encapsulated by the solid SNCs and ZNPs by adsorption. The solid particles aligned tightly to form a shell layer. The oil and water phases were separated by the shell layer because the solid molecules were firmly adsorbed at the oil–water interface, and fixed the oil droplet molecules. This enabled the close arrangement of the emulsion droplets without coalescence. This also verified the stabilization mechanism of Pickering emulsions, including the three-phase contact angle theory, the irreversible adsorption theory, and the mechanical barrier theory [35]. When the solid particles were in the right position, the Pickering emulsion was stabilized because droplet aggregation was prevented by volume exclusion and spatial resistance [36].

#### 3.5.3 Confocal laser scanning microscopy

The stability of the Pickering emulsion was related to the structure of the colloidal particles assembled at the interface. To investigate the interfacial shape of the menthol Pickering emulsion synergistically stabilized by ZNPs and SNCs, we stained the SNCs and ZNPs with Nile blue A and fluorescent brightener. Confocal laser scanning microscopy revealed blue and yellow particles. The confocal laser scanning microscopy plots of the menthol Pickering emulsion and the blank control are shown in Fig 6(D)–6(J). Our results reveal that the ZNPs were adsorbed on the oil-phase side and the SNCs were adsorbed on the water-phase side, and they formed a bilayer interfacial structure comprising two types of particles. The interfacial layer was thicker and was able to completely encapsulate the droplets and provide strength, which stabilized the Pickering emulsion. The bilayer colloidal particle interfaces comprised hydrophobic ZNPs on their interiors and hydrophilic SNCs on their exteriors. The properties of these particles were similar to those of Janus particles, so they were referred to as Janus interfaces. The high stability of the Pickering emulsion, which was synergistically stabilized by the ZNPs and SNCs, was attributed to the unique Janus interface [37].

### 3.6 Analysis of the emulsion rheological data

**Fig 7:**
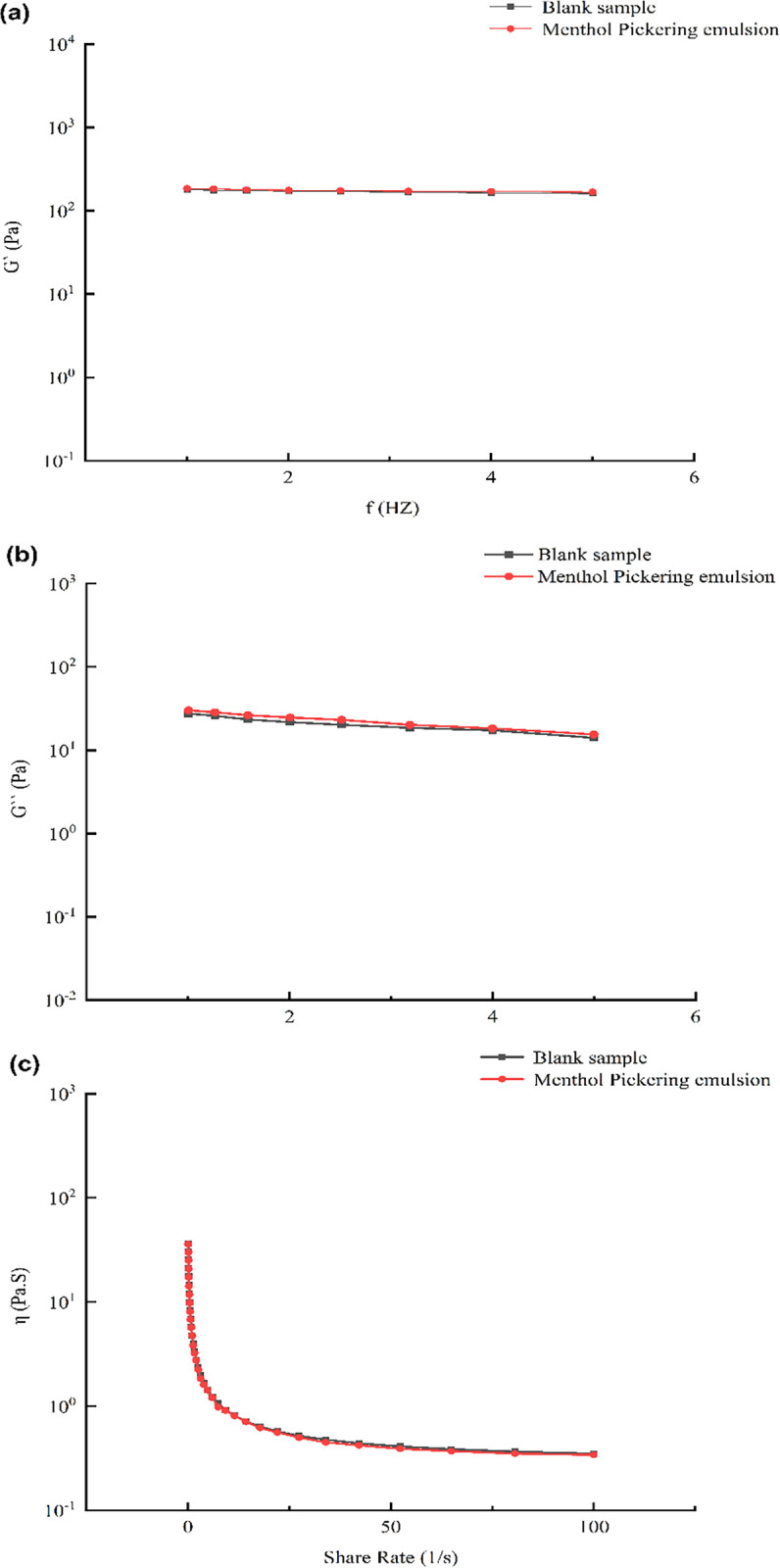
Rheological properties of Pickering emulsion, η: Apparent viscosity, Gʹ: Storage moduli, Gʹʹ: Loss moduli.

The rheological properties of the Pickering emulsion had an essential influence on its stability, and may have depended on its microstructure. Owing to the closer distance between the droplets of the Pickering emulsion, the rheological properties were significantly influenced by the stabilizer particles [38]. Fig 7 depicts the rheological results of a menthol Pickering emulsion optimized by the response surface experiment and the blank control. At a given concentration, the energy storage modulus of the Pickering emulsion was higher than the loss modulus, indicating that the elastic property was dominant; the emulsion exhibited a viscoelasticity similar to that of a gel. As shown in Fig 7(A) and 7(B), there was a slight difference in the G′ and G″ curves between the l-menthol-coated Pickering emulsion and the blank emulsion. This indicates that the stability of the Pickering emulsion was not affected by the l-menthol dissolved in the oil phase.

We also investigated the rheological properties of the menthol Pickering emulsion by determining its viscosity at various shear rates. Fig 7(C) reveals that the apparent densities of both the menthol Pickering emulsion and the blank Pickering emulsion decreased as the shear rate increased. This occurred because the integrity of the protective shell around the emulsion droplets and the network structure in the continuous phase were compromised at high shear rates. The results reveal that the emulsions typically exhibited shear thinning and pseudoplastic non-Newtonian fluid properties [39, 40].

**Fig 8:**
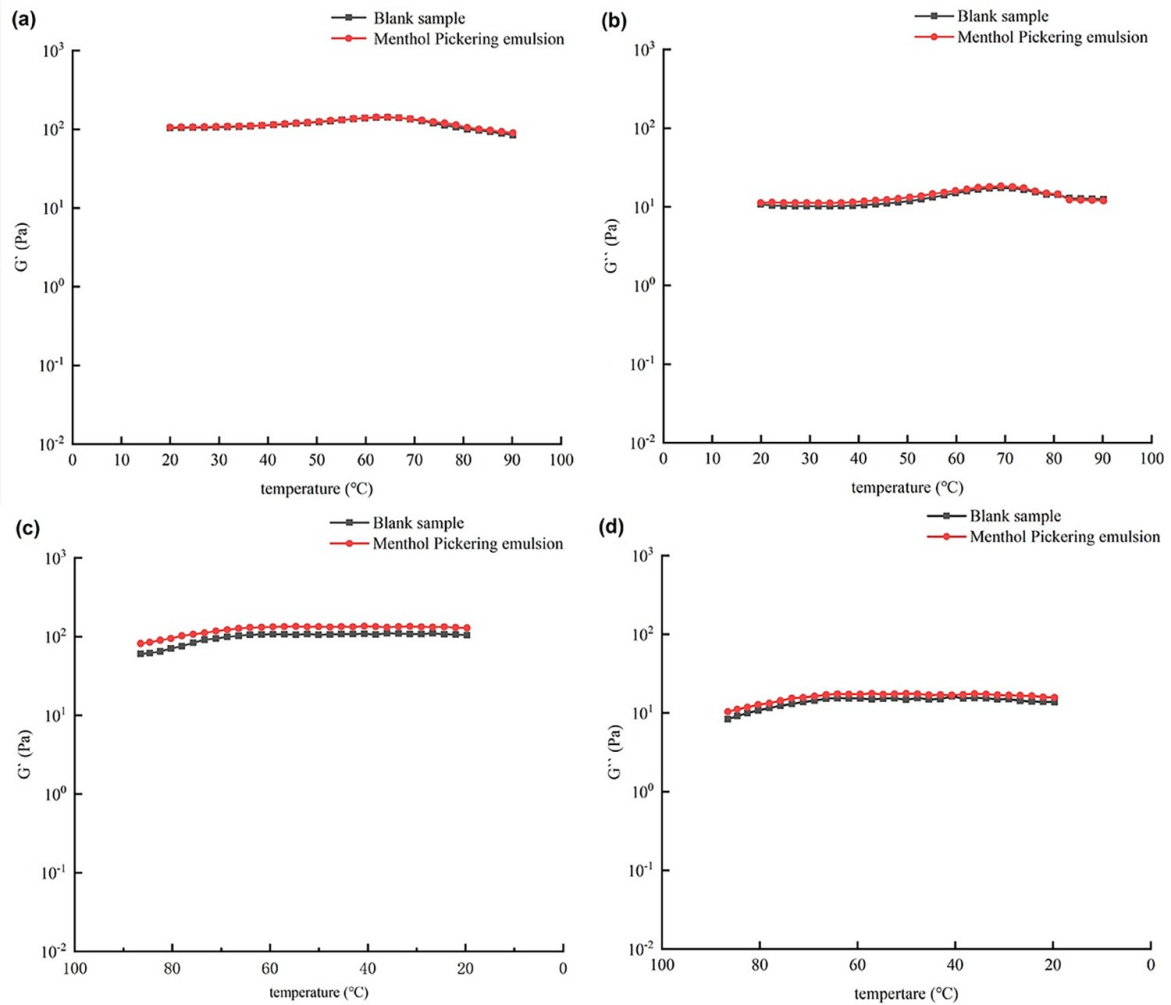
Effect of temperature change on the rheological properties of the blank and menthol Pickering emulsions, (a) and (b) were temperature rise plots of the menthol Pickering emulsion and the blank Pickering emulsion, (c) and (d) were temperature drop plots of the menthol Pickering emulsion and the blank Pickering emulsion.

Insight into the stability and microstructures of Pickering emulsions, which is key to their application, can be provided by their rheological properties [41]. The effect of temperature change on the rheological properties of the Pickering emulsions examined in the present study is shown in Fig 8. Throughout the temperature increase, the energy storage moduli of the emulsions were greater than their loss moduli. As the temperature increased, the energy storage modulus and loss modulus of each of the menthol and blank Pickering emulsions increased slowly over a particular range. This occurred because the heat treatment caused the protective shell of the solid particles at the oil–water interface of the emulsion and the network structure formed by the dispersed solid particles in the continuous phase to partially dissolve in water, leading to the transformation of the ordered initial system into a disordered state. The surface of the emulsion droplets roughened, resulting in increased intermolecular friction, which hindered the flow of the emulsion. However, the structure of the emulsion was not affected by this limited heat treatment. As the temperature continued to increase from 65 to 90°C, both the G′ and G″ values of the emulsion began to decrease. This may be attributed to the fact that the increase in temperature caused the molecules in the emulsion system to move violently and separate from the oil–water interface. However, as the temperature increased, there was a slight decrease in both the energy storage modulus and the loss modulus of the emulsion, indicating a partial disruption of its structure. This observation suggests that the emulsion exhibited some resilience to temperature change. This was because at high temperatures, an SNC provides a denser and stronger interface for Pickering emulsions, which improves their stability [42].

Fig 8(C) and 8(D) reveals that as the temperature decreased, the G′ and G″ values of the emulsion gradually increased. During this period, the stabilizer particles that were dissolved in the aqueous phase within the emulsion system were precipitated, and owing to the presence of intermolecular interactions, the damaged structure of the emulsion was repaired. As the temperature decreased to 20°C, the surfaces of the emulsion droplets changed from rough to smooth again, the partially disordered system became ordered again, and the G′ and G″ values of the emulsion slowly decreased.

### 3.7 Determination of the antioxidant capacity of the emulsion

The MDA contained in the oil phase is the end product of lipid peroxidation caused by free radicals, which are cytotoxic. Lipid peroxidation is the process by which oxidants such as free radicals attack lipids containing unsaturated fatty acids, producing lipid peroxyl radicals and hydroperoxides [43]. The MDA content is an important parameter because it reflects the antioxidant capacity of an emulsion, and can indicate the rate and intensity of lipid peroxidation in the emulsion [44]. Therefore, the MDA content of an emulsion indicates its degree of oxidation and consequently its safety within the human body.

**Fig 9:**
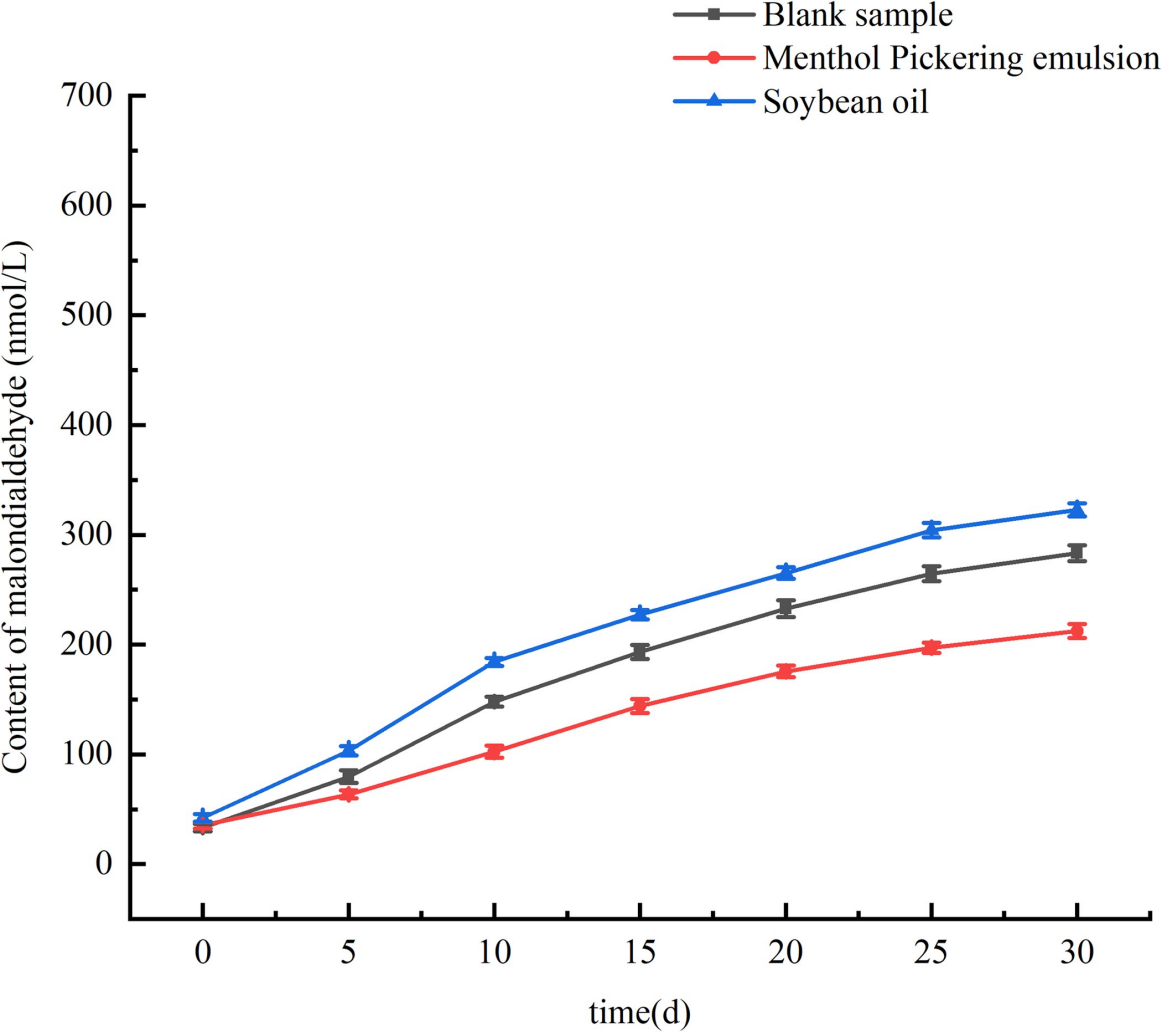
The variation trend of MDA content in the blank Pickering emulsion,the mentol Pickering emulsion and Soubean oil with storage time.

Fig 9 shows the MDA contents of the soybean oil, the blank emulsion, and the menthol Pickering emulsion over the storage period. The MDA content of the menthol Pickering emulsion was 212.4 nmol/L, which was lower than the MDA contents of both the blank emulsion (283.34 nmol/L) and the soybean oil (322.7 nmol/L). In the menthol Pickering emulsions stabilized by ZNCs and ZNPs, the solid particles were adsorbed at the oil–water interface to form a dense protective shell, which effectively isolated the external oxygen and slowed down the oxidation of the oils in the emulsions. The menthol Pickering emulsions had the lowest MDA contents. This may be attributed to the fact that l-menthol is an active agent with antioxidant properties [45–47]. Loading an emulsion with l-menthol creates an antioxidant interface that effectively protects the lipids and delays lipid oxidation in an emulsion [48].

### 3.8 *In vitro* digestive simulation

**Fig 10:**
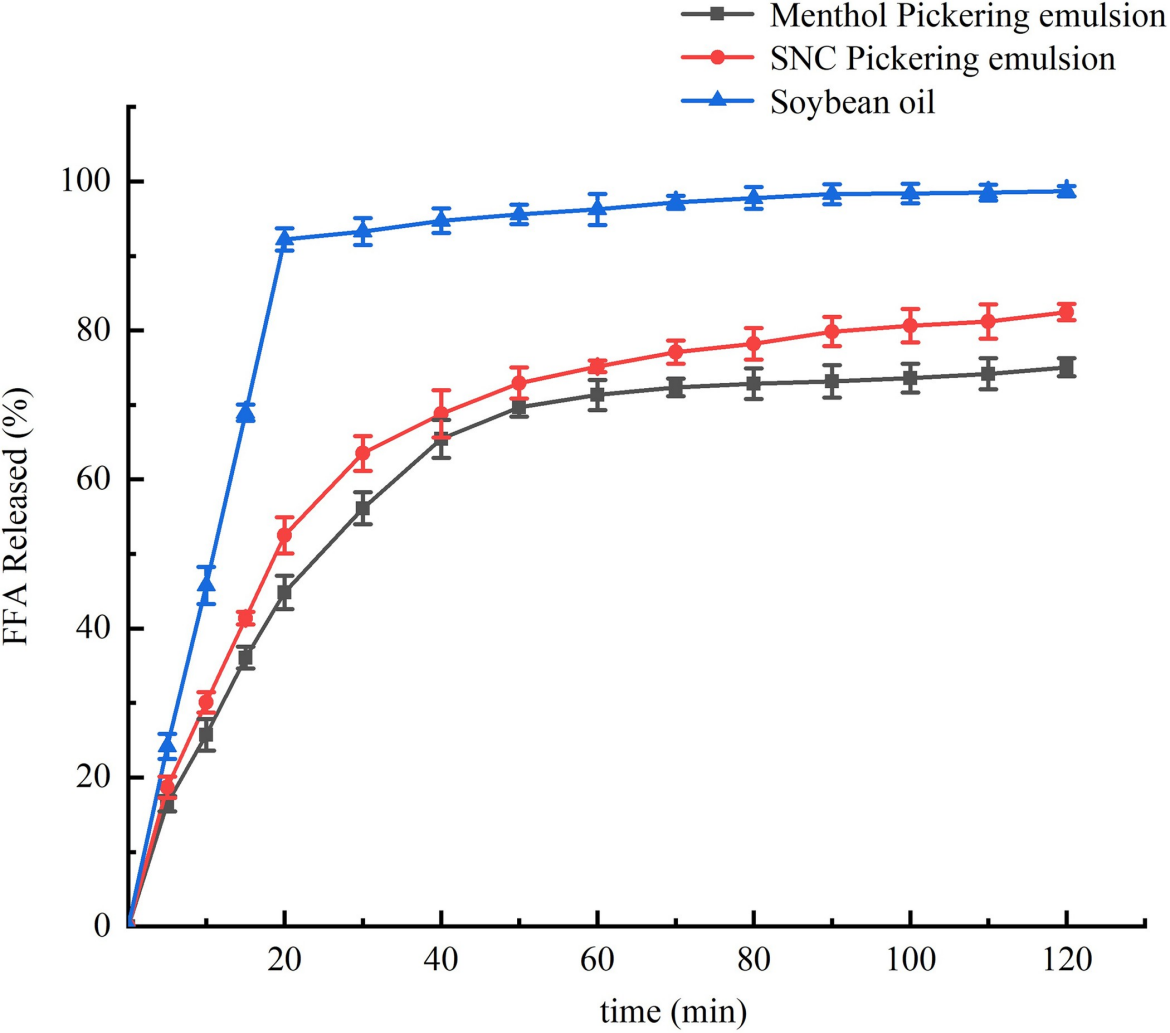
Release rate of FFA from Pickering emulsion in an in vitro simulated digestion model.

The pH-stat method [18] is used to determine the rate of free fatty acid release from emulsions during digestion. Pickering emulsions prepared from SNCs and soybean oil were chosen as the blank control, and the results are shown in Fig 10. There was a rapid increase in the free fatty acid content of the emulsions during the first 30 min of digestion. This was owing to the fact that the fats were broken down during the initial stages of digestion, producing large amounts of glycerol and free fatty acids. After 120 min of simulated small intestinal digestion, the free fatty acid release rate of the menthol Pickering emulsion synergistically stabilized by ZNPs and SNCs was 75.06 ± 1.23%, which was lower than that of the Pickering emulsion stabilized by SNCs alone (83.46 ± 1.08%). This indicates that the interfacial layer formed by the ZNPs and SNCs had good barrier properties and effectively resisted and slowed down the hydrolysis of digestive enzymes, thereby slowing down the degradation of fats and oils. Emulsion systems have been used in a variety of industries such as food, agriculture, detergents, and cosmetics [49], and the study of the *in vitro* simulated digestion characteristics of emulsions may help to expand the application of emulsions in the food sector.

### 3.9 Bacteriostatic testing of the Pickering emulsion

Significant antibacterial and antifungal activity has been reported in gram-positive and gram-negative bacteria, as well as yeasts and fungi, when exposed to peppermint essential oil. This activity is mainly attributable to the main chemical constituents of the oil, i.e., l-menthol and menthone [50]. However, the antibacterial activity of l-menthol is greatly reduced by its poor water solubility and volatility.

**Fig 11:**
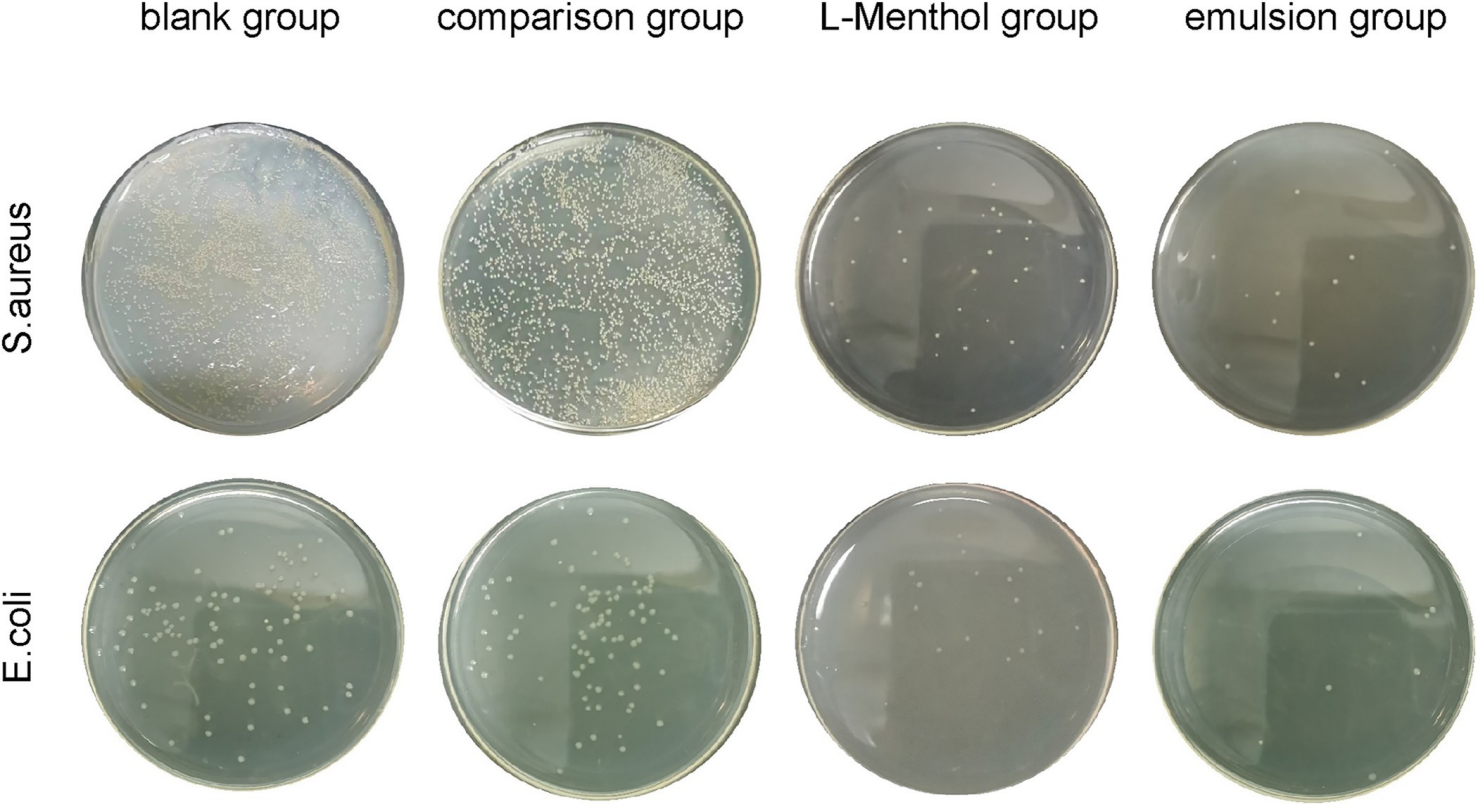
Plate colony diagrams of different samples.

The delivery of target substances via o/w emulsion ternary systems has been reported frequently. For example, Siddique et al. [51] developed a μE formulation comprising clove oil, Brij-35, isopropanol, and water for loading into water-soluble fluoroquinolone antibiotics. Saleem et al. [52] developed an o/w microemulsion formulation and successfully prepared nanoparticles of fluoroquinolone antibiotics, which demonstrated a significant increase in the antibacterial activity of the nanoparticles. To investigate the protective effect of an l-menthol Pickering emulsion on the bacteriostatic efficacy of l-menthol, common *E*. *coli* and *S*. *aureus* were selected for bacteriostatic testing according to the method described by Zhao et al. [53]. The minimum inhibitory concentration of l-menthol with regard to *E*. *coli* and *S*. *aureus* was 48 ± 0.08 μg/mL and 86 ± 0.03 μg/mL, respectively. Therefore, 1 mL of an l-menthol Pickering emulsion with a concentration of 40 μg/mL and an equivalent mass of l-menthol crystals was selected as the drug-added group. The final results are shown in Fig 11. In the *S*. *aureus* group, the total numbers of colonies were approximately 2.5 × 10^9^ CFU/mL in the blank and control groups, 2 × 10^8^ CFU/mL in the l-menthol group, and 1 × 10^8^ CFU/mL in the menthol Pickering emulsion group. In the *E*. *coli* group, the total numbers of colonies were approximately 6 × 10^7^ CFU/mL in the blank and control groups, 1.2 × 10^7^ CFU/mL in the l-menthol group, and 6 × 10^6^ CFU/mL in the menthol Pickering emulsion group.

Based on the experimental results, the Pickering emulsion without embedded l-menthol had no effect on the total number of colonies. The total number of colonies in the l-menthol group was greater than that in the menthol Pickering emulsion group in all cases, indicating that the Pickering emulsion had a protective effect on l-menthol. This protective effect allowed the antibacterial efficacy of l-menthol to be fully utilized.

## 4. Conclusions

In the present experiment, L-menthol was successfully embedded in a Pickering emulsion that had been synergistically stabilized with ZNPs and SNCs. Response surface experiments optimized the ratio of ZNPs to SNCs at 1:1, with a particle concentration of 2 wt%, an oil-to-water ratio of 1:1, and an embedding rate of 83%. The Pickering emulsion was exceptionally stable over 30 days and gradually released menthol while maintaining bioavailability. It had excellent rheological and antioxidant properties, and a low MDA content (212.407 nmol/g) during storage. Bacteriostatic evaluations indicated superior inhibition of *S*. *aureus* and *E*. *coli*. The present study will extend the applicability of l-menthol and provides a theoretical basis for the encapsulation of other active ingredients for use in the food industry.

## Supporting information

S1 Text The formula of the membership degree and composite score. (DOCX)

S2 Text The formula of the L-menthol embedding rate of the Pickering emulsion. (DOCX)

S3 Text The formula of the FFA release rate. (DOCX)

S1 Graphical abstract (TIF)
