# Supplementary material for: Synergistic stabilization of a menthol Pickering emulsion by zein nanoparticles and starch nanocrystals: Preparation, structural characterization, and functional properties
Source: PLoS One. 2024 Jun 6;19(6):e0303964. doi: 10.1371/journal.pone.0303964 (PMC11156346; doi:10.1371/journal.pone.0303964)
Supplement: S1 Text — (DOCX) [file pone.0303964.s001.docx]

**Formula (1), (2), (3)**

$$\begin{aligned} P=\frac{A_{max}-A_{i}}{A_{max}-A_{min}}\#\left( 1 \right) \end{aligned}$$

$$\begin{aligned} P=\frac{A_{i}-A_{min}}{A_{max}-A_{min}}\#\left( 2 \right) \end{aligned}$$

$$\begin{aligned} S=a_{1}P_{1}+a_{2}P_{2}+a_{3}P_{3}\#\left( 3 \right) \end{aligned}$$

A_max_ — the maximum value of each indicator factor, A_min_ —the minimum value of each indicator factor, A_i_ — the value of a factor of the indicator. S —Composite score; a_1_, a_2_, a_3_—weight of emulsification value, potential, embedding rate; P_1_, P_2_, P_3_—membership of emulsification value, potential, embedding rate
