# Supplementary material for: Synergistic stabilization of a menthol Pickering emulsion by zein nanoparticles and starch nanocrystals: Preparation, structural characterization, and functional properties
Source: PLoS One. 2024 Jun 6;19(6):e0303964. doi: 10.1371/journal.pone.0303964 (PMC11156346; doi:10.1371/journal.pone.0303964)
Supplement: S2 Text — (DOCX) [file pone.0303964.s002.docx]

**Formula (4)**

$$\begin{aligned} Embedding rate=\frac{Embedding L-Menthol content}{Total L-Menthol content}\#\left( 4 \right) \end{aligned}$$
