# Supplementary material for: Synergistic stabilization of a menthol Pickering emulsion by zein nanoparticles and starch nanocrystals: Preparation, structural characterization, and functional properties
Source: PLoS One. 2024 Jun 6;19(6):e0303964. doi: 10.1371/journal.pone.0303964 (PMC11156346; doi:10.1371/journal.pone.0303964)
Supplement: S3 Text — (DOCX) [file pone.0303964.s003.docx]

**Formula (5)**

The calculation formula is given below:

$$\begin{aligned} FFA Realeased\%=\frac{100\times V_{NaOH}\times C_{NaOH}}{2M_{trigliycerides}}\#\left( 5 \right) \end{aligned}$$

where Mtrigliyce fourides is the average molecular mass of fat (g/mol); V_NaOH_ is the volume of NaOH solution consumed at simulated small intestinal digestion time t (mL); and C_NaOH_ is the concentration of the standard NaOH solution used for titration (mol/L)
