# Supplementary figures and images for: Synergistic stabilization of a menthol Pickering emulsion by zein nanoparticles and starch nanocrystals: Preparation, structural characterization, and functional properties
Source: PLoS One. 2024 Jun 6;19(6):e0303964. doi: 10.1371/journal.pone.0303964 (PMC11156346; doi:10.1371/journal.pone.0303964)

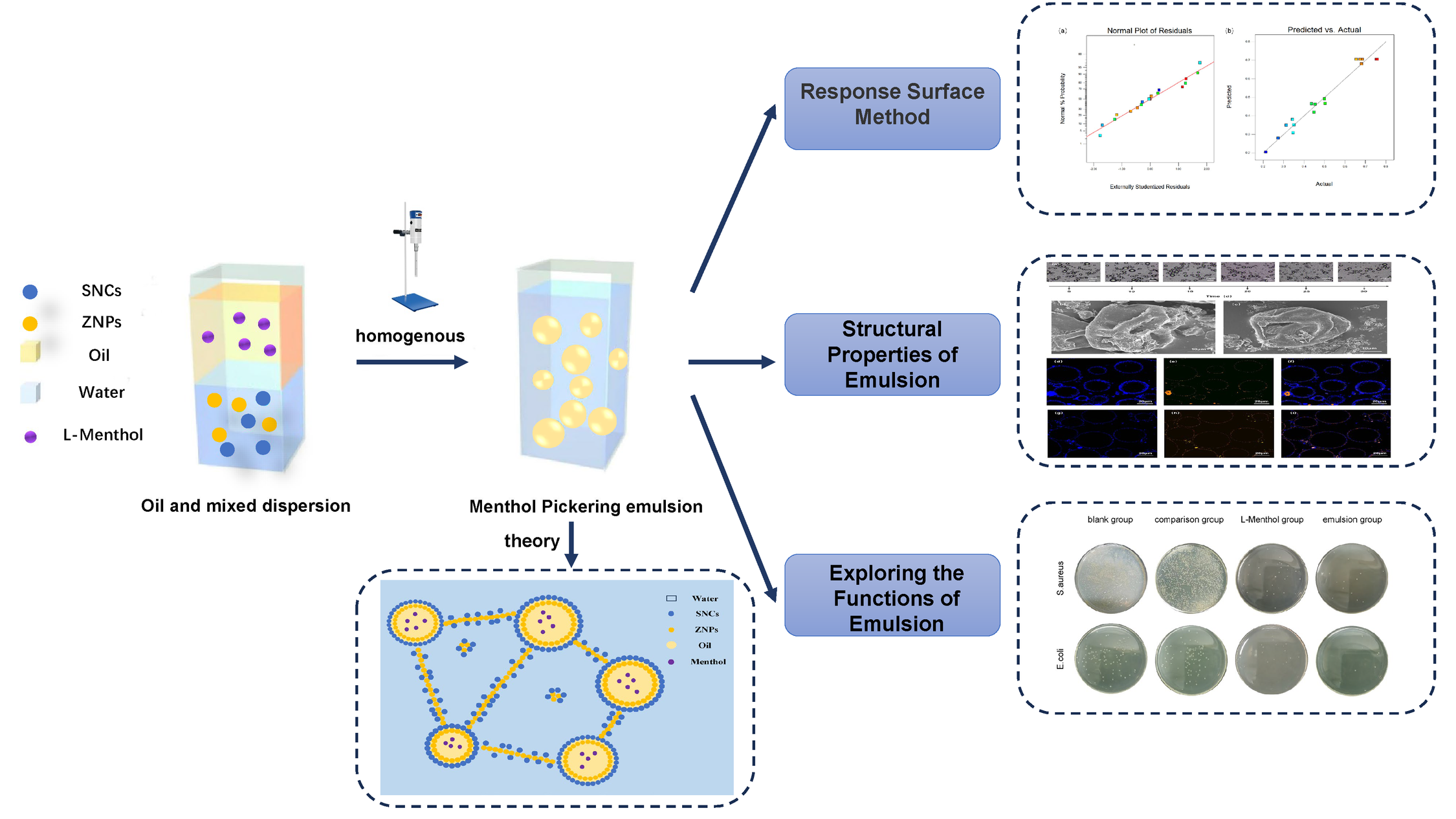

Supplement: S1 Graphical abstract — (TIF) [file pone.0303964.s004.tif]
